# Assessment of Racial Disparities in Mortality Rates Among Older Adults Living in US Rural vs Urban Counties From 1968 to 2016

**DOI:** 10.1001/jamanetworkopen.2020.12241

**Published:** 2020-08-03

**Authors:** Nasim B. Ferdows, María P. Aranda, Julie A. Baldwin, Soroosh Baghban Ferdows, Jasjit S. Ahluwalia, Amit Kumar

**Affiliations:** 1 Department of Health Administration and Policy, Hudson College of Public Health, University of Oklahoma Health Sciences Center, Oklahoma City; 2 Edward R. Roybal Institute on Aging, Suzanne Dworak-Peck School of Social Work, University of Southern California, Los Angeles; 3 Center for Health Equity Research, Northern Arizona University, Flagstaff; 4 Istanbul Technical University, Istanbul, Turkey; 5 Center for Alcohol and Addiction Studies, Department of Behavioral and Social Sciences, Brown University School of Public Health, Providence, Rhode Island; 6 College of Health and Human Services, Northern Arizona University, Flagstaff

## Abstract

**Importance:**

Population-based mortality rates are important indicators of overall health status. Mortality rates may reflect underlying disparities in access to health care, quality of care, racial and geographical variations, and other socioeconomic factors associated with health. However, there is limited information on historical trends in mortality rates between older Black and White adults living in urban compared with rural communities.

**Objective:**

To examine historical trends of mortality rates among White adults compared with Black adults and among rural residents compared with urban residents by comparing sex-specific age-adjusted all-cause mortality rates between older adults of both races who reside in rural and urban counties in the US.

**Design, Setting, and Participants:**

In this county-level cross-sectional longitudinal study of US counties from January 1, 1968, to December 31, 2016, mortality data were obtained from the CDC WONDER database of the Centers for Disease Control and Prevention, and socioeconomic characteristics were obtained from the Area Health Resources Files of the US Health Resources and Services Administration. The study population included older adults (≥65 years) of Black and White ancestry living in 3131 rural and urban counties in the US. Using ordinary least squares regression analyses, race- and sex-specific trends in mortality rates with 95% CIs were examined, and trends adjusted by county-level socioeconomic characteristics using year and county fixed-effects were calculated. Data were analyzed from March 24 to May 10, 2020.

**Exposures:**

Three geographic regions were examined: urban counties, rural counties adjacent to an urban county (rural-adjacent counties), and rural counties not adjacent to an urban county (rural-nonadjacent counties).

**Main Outcomes and Measures:**

All-cause age-adjusted mortality rates of Black and White adults 65 years and older.

**Results:**

For 1968, a total of 3076 counties (19 240 437 adults ≥65 years; 11 100 000 women [57.69%]; 1 484 747 Black individuals [7.74%]) were identified; of those, 1138 counties were urban, 1018 counties were rural adjacent, and 922 counties were rural nonadjacent. For 2016, a total of 3087 counties (46 400 000 adults ≥65 years; 25 800 000 women [55.72%]; 4 447 733 Black individuals [9.60%]) were identified; of those, 1163 counties were urban, 1020 counties were rural adjacent, and 904 counties were rural nonadjacent. Between 1968 and 2016, mortality rates per 100 000 persons decreased from 9063 to 4896 deaths (46%) among White men and from 6175 to 3760 deaths (39%) among White women. During the same period, mortality rates decreased from 8801 to 5477 deaths (38%) among Black men and from 6380 to 3960 deaths (38%) among Black women. However, the racial mortality gap increased among men living in rural counties after 1980. From 1968 to 2016, the mortality rate among White men decreased from 9063 to 4751 deaths (48%) in urban counties, from 9113 to 5338 deaths (41%) in rural-adjacent counties, and from 8971 to 5229 deaths (42%) in rural-nonadjacent counties. The mortality rate among Black men during the same period decreased from 8715 to 5368 deaths (38%) in urban counties, from 8924 to 6458 deaths (28%) in rural-adjacent counties, and from 9500 to 6941 deaths (27%) in rural-nonadjacent counties.

**Conclusions and Relevance:**

Rural and urban socioeconomic differences were associated with mortality rate disparities among both White and Black women. However, rural vs urban disparities in mortality rates among men remained significant, especially among Black men living in rural counties. Notably, the current mortality rate of Black men living in rural areas is similar to that of White men living in urban and rural areas in the mid-1980s. Understanding the intersectional factors associated with health disparities may help to inform public health and clinical interventions.

## Key Points

**Question:**

Do historical trends exist in age-adjusted mortality rates among older adults of Black and White ancestry living in rural and urban counties in the US?

**Findings:**

In this cross-sectional study of 3131 US counties over a 49-year period, racial disparities in the mortality rates of men, with the exception of men living in rural counties, decreased compared with women. Rural and urban disparities were associated with socioeconomic differences among men and women of both races, and these disparities were most substantial among Black men.

**Meaning:**

Although the overall trend in the differences among Black and White older adults’ mortality rate have narrowed in the urban areas, there is considerable widening in mortality rate among Black and White men living in rural counties.

## Teaser

This cross-sectional study uses data from the CDC WONDER database and the Area Health Resources Files to examine historical trends and disparities in mortality rates between Black and White adults 65 years and older living in urban and rural communities in the US from 1968 to 2016.

## Introduction

Over the past century, mortality rates among older adults in the US have substantially decreased among both Black and White individuals. In recent years, the historical racial disparity gap has also decreased, reflecting a greater reduction in mortality rates among Black adults in almost every age group.^1,2^ Studies of geographical disparities in mortality among all age groups have indicated greater mortality decreases among individuals living in urban compared with rural communities over the past 2 decades and a greater decrease in mortality among White adults living in urban areas.^3^ Some studies have reported mortality differences according to racial,^1,2^ socioeconomic,^1,2^ or geographic factors for the general US population.^3^ However, there is limited information on historical trends in mortality rates between older Black and White adults living in urban compared with rural communities. Data suggest that rural residents experience considerable disparities in disease, morbidity, preventable deaths, longevity, life expectancy, and mortality compared with their urban counterparts.^4,5^

Because mortality rates measure the health status of a given population, the estimation of mortality rates in urban and rural areas may identify health disparities, guide health care resource allocation, and inform clinical and population-based care interventions. The objective of this study was to compare the mortality rate trends of older Black and White adults living in rural and urban communities over almost 50 years. To provide a comprehensive overview of geographic differences in US mortality rates, we investigated sex-specific age-adjusted mortality rates of older Black and White adults living in US rural and urban counties. Insights gained from this study can inform efforts aimed at improving health equity in rural areas.

## Methods

Using mortality data from the CDC WONDER database from January 1, 1968, to December 31, 2016, we analyzed the trends in age-adjusted mortality rates of individuals 65 years and older across 3131 US counties. The CDC WONDER database contains county-level national mortality and population data collected by the National Center for Health Statistics of the Centers for Disease Control and Prevention.^6,7,8^ We added data from the Area Health Resources Files of the US Health Resources and Services Administration between 1992 and 2014 to include county-level socioeconomic characteristics, including per capita income, unemployment rates, and poverty rates. We analyzed the trends in age-adjusted all-cause mortality rates of the population 65 years and older, adjusting for county-level characteristics to eliminate differences in county characteristics. Because this study used publicly available county-level national data, in which no individual information would be recognizable, the study is exempt from institutional review board approval according to the policies of Brown University’s institutional review board and informed patient consent owing to the infeasibility of acquiring consent in claims data. This study followed the Strengthening the Reporting of Observational Studies in Epidemiology (STROBE) reporting guideline for cross-sectional studies.

We used the 2013 Rural-Urban Continuum Codes from the US Department of Agriculture Economic Research Service^9^ to categorize a pooled number of counties from 1968 to 2016 by level of urbanization: urban counties (37%), rural counties adjacent to an urban county (rural-adjacent counties; 33%), and rural counties not adjacent to an urban county (rural-nonadjacent counties; 30%). Among 3143 total US counties, 93 counties were not included in the 2013 Rural-Urban Continuum Codes. Of those, 70 counties were captured by searching 2003 continuum codes, and 23 counties were captured in 1993 continuum codes. Counties with unreliable mortality rates were excluded, with unreliable mortality rate defined as a rate with a numerator of 20 or less. The final sample comprised 3131 US counties in 50 states and the District of Columbia from 1968 to 2016.

The outcome measured was the county-level age-adjusted all-cause mortality rates of Black and White adults 65 years and older. The age-adjusted mortality rate was defined as the weighted average of age-specific mortality rates, in which the weighted values represent a fixed population by age. Rates were calculated per 100 000 persons. We used age-adjusted rates to eliminate variations that may have occurred owing to age distribution differences in certain populations and to compare relative mortality risk between groups over time. The age-adjusted mortality rates were calculated for each race and sex group per county in a given year. The racial categories included White and Black, as reported on death certificates in accordance with standards from the US Office of Management and Budget.^6,7,8^

We also accounted for several socioeconomic factors, such as unemployment rates and per capita income using data from the Area Health Resources Files for each county by year. Missing values were imputed for per capita income using the mean value of the observed variables (eg, 2915 observations were imputed for 2009).

## Statistical Analysis

First, we performed ordinary least squares regression analyses of the mortality and year interaction with race, sex, and rurality to depict age- and sex-specific trends in mortality by level of rurality. To describe changes in rural and urban mortality over time, we estimated the trends in race- and sex-specific rural vs urban (rural-urban) mortality gaps using fixed-effects regression for year weighted by county population, and we reported trends and 95% CIs. The 95% CIs described the variations in outcome during each study period. Second, we performed ordinary least squares regression analyses using fixed-effects regression for year and state, controlled for county-level per capita income and unemployment rates from 1992 to 2014. Using fixed-effects regression analyses for state, we accounted for unobserved time-invariant differences between states that may have been associated with trends. Data analysis was performed using Stata software, version 15 (StataCorp), and data were analyzed from March 24 to May 10, 2020.

## Results

For 1968, we identified 3076 counties in 49 US states and the District of Columbia (19 240 437 adults ≥65 years; 11 100 000 women [57.69%]; 1 484 747 Black individuals [7.74%]; Alaska was not included in 1968 records); of those, 1138 counties were urban, 1018 counties were rural adjacent, and 922 counties were rural nonadjacent. For 2016, we identified 3087 counties in 50 states and the District of Columbia (46 400 000 adults ≥65 years; 25 800 000 women [55.72%]; 4 447 733 Black individuals [9.60%]); of those, 1163 counties were urban, 1020 counties were rural adjacent, and 904 counties were rural nonadjacent.

Compared with urban counties, between 1992 and 2014, rural-adjacent and rural-nonadjacent counties had lower per capita income (in 1992, $20 981 in urban counties vs $15 545 and $15 604 in rural-adjacent and rural-nonadjacent counties, respectively; in 2014, $47 164 in urban counties vs $36 234 and $38 233 in rural-adjacent and rural-nonadjacent counties), higher unemployment rates (in 1992, 7.49% in urban counties vs 8.33% and 7.76% in rural-adjacent and rural-nonadjacent counties; in 2014, 6.23% in urban counties vs 6.71% and 6.57% in rural-adjacent and rural-nonadjacent counties), lower median household income (in 1993, $32 076 in urban counties vs $24 982 and $24 457 in rural-adjacent and rural nonadjacent counties; in 2014, $53 527 in urban counties vs $41 777 and $42 783 in rural-adjacent and rural nonadjacent counties), higher poverty rates (in 1997, 13.02% in urban counties vs 16.99% and 17.11% in rural-adjacent and rural nonadjacent counties), a higher proportion of adults 65 years and older (in 1994, 12.18% in urban counties vs 15.07% and 15.30% in rural-adjacent and rural-nonadjacent counties; in 2014, 13.96% in urban counties vs 17.80% and 17.66% in rural-adjacent and rural-nonadjacent counties), and less diversity (in 1992, 8.59% of individuals in urban counties were Black vs 6.06% and 4.35% of individuals in rural-adjacent and rural-nonadjacent counties; in 2014, 10.47% of individuals in urban counties were Black vs 5.42% and 3.71% of individuals in rural-adjacent and rural-nonadjacent counties) (eTable, eFigure 1, and eFigure 2 in the Supplement).

**Figure 1.**
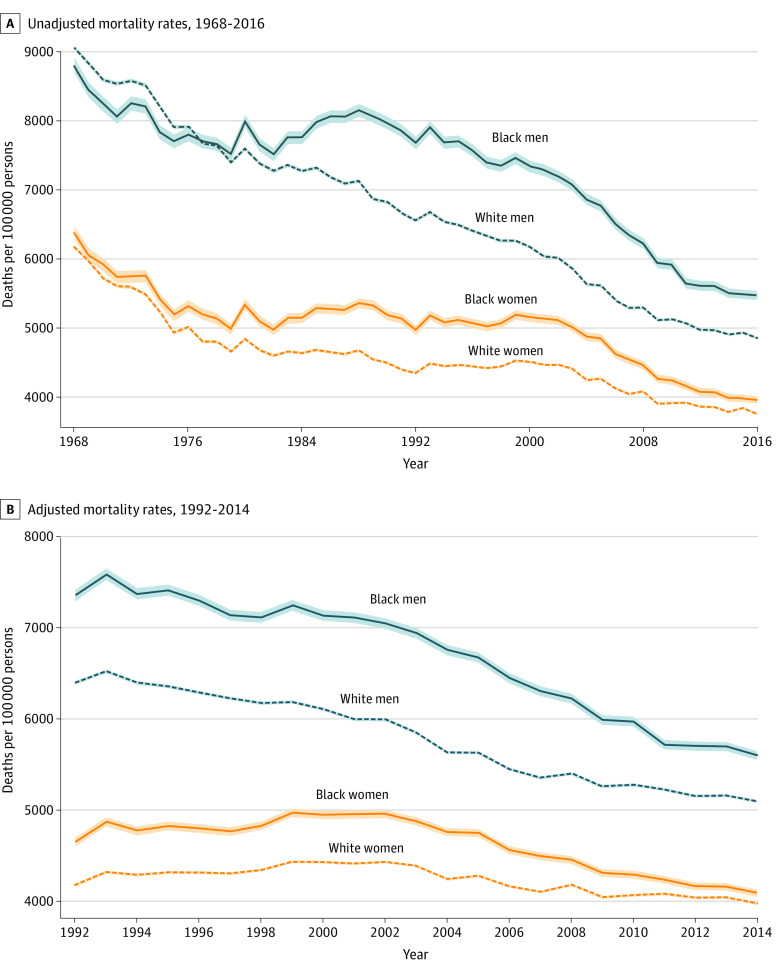
Race-Specific and Sex-Specific Age-Adjusted All-Cause Mortality Rates in Individuals 65 Years and Older The data for all-cause mortality rates and county-level population characteristics were obtained from the CDC Wonder Compressed Mortality Files. The data for county-level per capita income and unemployment rates were obtained from the Area Health Resources Files. Plots were weighted by county population. Shaded areas represent 95% CIs. A, Unadjusted mortality rates, 1968-2016. B, Adjusted mortality rates, 1992-2014. Adjusted for county-level income and unemployment rate.

From 1968 to 2016, mortality rates decreased for both sexes (Figure 1A). The age-adjusted mortality rate per 100 000 people decreased from 9063 to 4896 deaths (46%) among White men and from 6175 to 3760 deaths (39%) among White women. Mortality rates decreased from 8801 to 5477 deaths (38%) among Black men and from 6380 to 3960 deaths (38%) among Black women. After controlling for county-level socioeconomic characteristics, the greatest decreases in mortality rates occurred among Black men (from 7361 deaths to 5604 deaths [24%]) followed by White men (from 6403 deaths to 5099 deaths [20%]), Black women (from 4653 deaths to 4090 deaths [12%]), and White women (from 4178 deaths to 3976 deaths [5%]) (Figure 1B).

### Rural and Urban Racial Disparities

**Figure 2.**
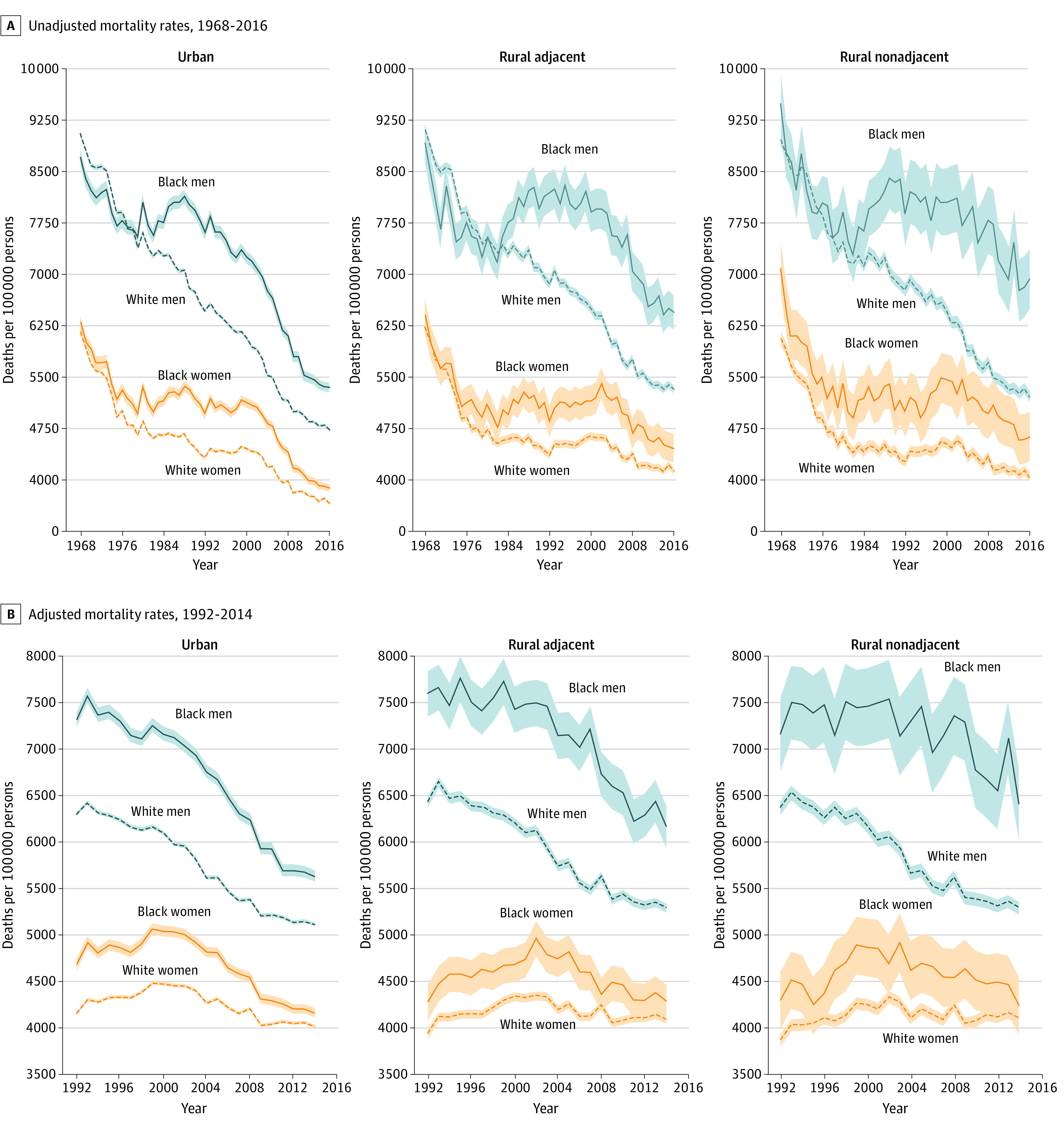
Racial Disparities Among Individuals 65 Years and Older in Rural vs Urban US Counties The data for all-cause mortality rates and county-level population characteristics were obtained from the CDC Wonder Compressed Mortality Files. The data for county-level per capita income and unemployment rates were obtained from the Area Health Resources Files. Plots were weighted by county population. Shaded areas represent 95% CIs. A, Unadjusted mortality rates, 1968-2016. B, Adjusted mortality rates, 1992-2014. Adjusted for county-level income and unemployment rate.

During the first decade examined (1968 to 1979), no mortality gap was observed between Black and White adults within each rural and urban category (Figure 2A). In urban counties, the mortality rate decreased from 9063 to 7394 deaths (18%) for White men, from 8716 to 7550 deaths (13%) for Black men, from 6172 to 4677 deaths (24%) for White women, and from 6314 to 4988 deaths (21%) for Black women. In rural-adjacent counties, the mortality rate decreased from 9113 to 7453 deaths (18%) for White men, from 8924 to 7263 deaths (17%) for Black men, from 6249 to 4655 deaths (26%) for White women, and from 6425 to 4929 deaths (23%) for Black women. In rural-nonadjacent counties, the mortality rate decreased from 8972 to 7349 deaths (18%) for White men, from 9500 to 7620 deaths (20%) for Black men, from 6973 to 4536 deaths (25%) for White women, and from 7099 to 5061 deaths (29%) for Black women.

The racial gap, which started in the early 1980s, increased during the following 20 years. Compared with White men, approximately 1200 more deaths per 100 000 Black men occurred in urban counties in 2004, and 1900 more deaths per 100 000 Black men occurred in rural-adjacent areas in 2007. Compared with White women, approximately 650 more deaths per 100 000 Black women occurred in urban areas, and 782 more deaths per 100 000 Black women occurred in rural-adjacent counties in 2002. The racial gap began to decrease in the mid-2000s in urban and rural-adjacent counties but remained greater in rural-nonadjacent counties (8100 more deaths per 100 000 Black men compared with White men and 960 more deaths per 100 000 Black women compared with White women). However, the mortality gap between Black and White men decreased only in urban counties and increased in both rural-adjacent and rural-nonadjacent counties (Figure 2A).

The 95% CIs, shown as shaded areas in Figure 2B, indicated that there was more variability in the mortality rates of Black adults in rural areas. After controlling for county-level socioeconomic characteristics, the racial gap remained highest and continued to increase in rural-nonadjacent counties (1122 more deaths per 100 000 Black men compared with White men).

### Rural and Urban Disparities

**Figure 3.**
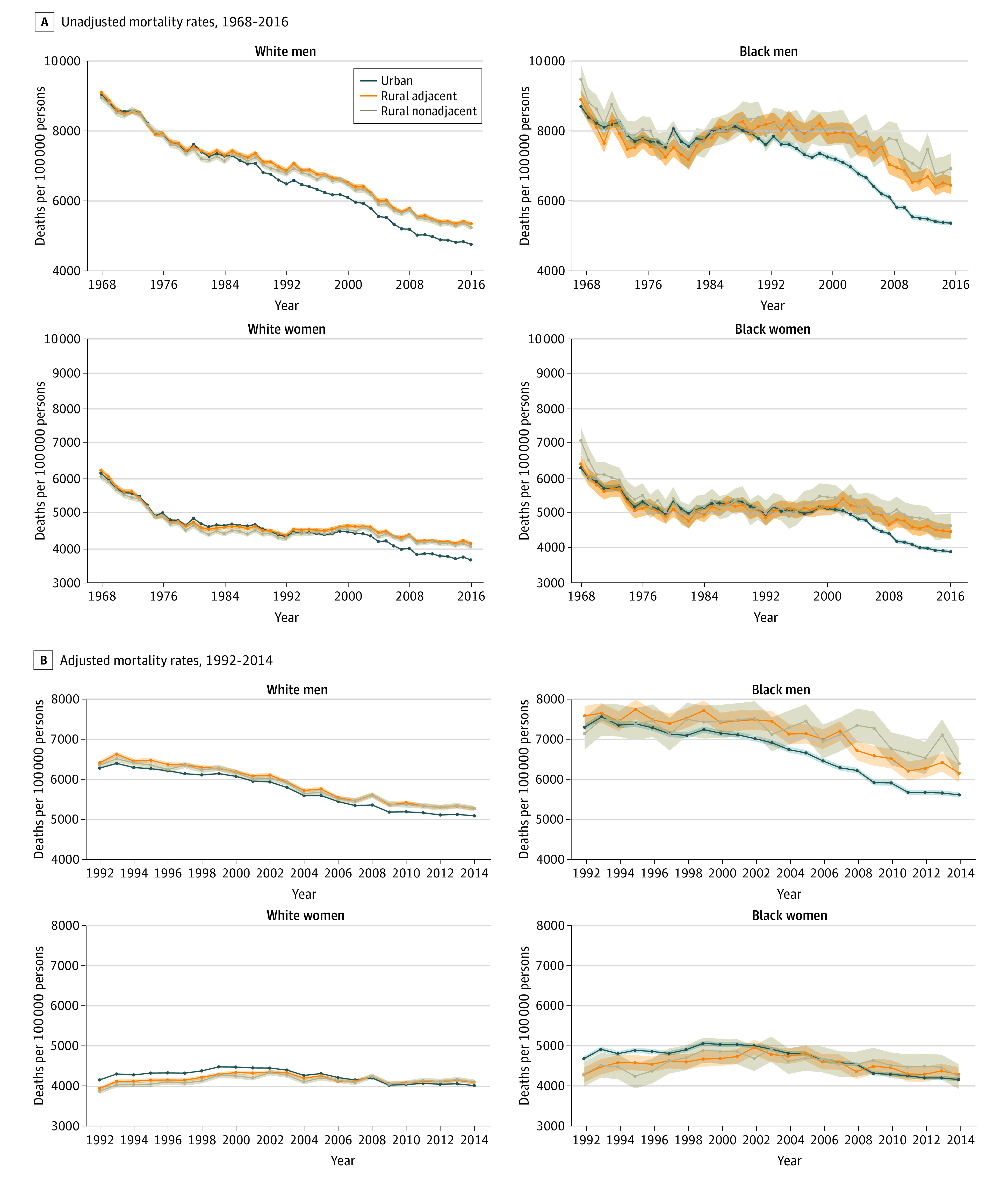
Rural and Urban Disparities in Race-Specific and Sex-Specific All-Cause Mortality Rates in Individuals 65 Years and Older The data for all-cause mortality rates and county-level population characteristics were obtained from the CDC Wonder Compressed Mortality Files. The data for county-level per capita income and unemployment rates were obtained from the Area Health Resources Files. Plots were weighted by county population. Shaded areas represent 95% CIs. A, Unadjusted mortality rates, 1968-2016. B, Adjusted mortality rates, 1992-2014. Adjusted for county-level income and unemployment rate.

During the first 2 decades examined, no rural-urban mortality gap between race and sex groups was found (Figure 3A). Beginning in the mid-1980s, the mortality rates of White men in urban areas decreased more than that of White men in rural areas, creating a rural-urban mortality gap that has continued to increase. However, among Black men, the rural-urban mortality gap started in the late 1980s and increased over time. After controlling for county-level socioeconomic characteristics, the mortality gap in rural compared with urban counties persisted among men of both races but was more substantial, and continued to increase, among Black men (Figure 3B).

From 1968 to 2016, mortality rates among White men decreased from 9063 to 4751 deaths (48%) in urban counties, from 9113 to 5338 deaths (41%) in rural-adjacent counties, and from 8971 to 5229 deaths (42%) in rural-nonadjacent counties. Among Black men, the rates decreased from 8715 to 5368 deaths (38%) in urban counties, from 8924 to 6458 deaths (28%) in rural-adjacent counties, and from 9500 to 6941 deaths (27%) in rural-nonadjacent counties. Among White women, the mortality rates decreased from 6172 to 3679 deaths (40%) in urban counties, from 6249 to 4150 deaths (34%) in rural-adjacent counties, and from 6073 to 4063 deaths (33%) in rural-nonadjacent counties. Among Black women, the mortality rates decreased from 6313 to 3907 deaths (38%) in urban counties, from 6425 to 4482 deaths (30%) in rural-adjacent counties, and from 7099 to 4649 deaths (35%) in rural-nonadjacent counties.

Comparing the 2 rural settings with the urban setting, the rural-urban mortality gap increased substantially among Black men over time; this increase was significantly higher than that of women and White adults from the mid-1990s. This gap remained significant for older Black men after controlling for county-level socioeconomic characteristics (eFigure 3 in the Supplement).

## Discussion

The racial mortality gap has varied from 1968 to 2016. During the earlier period, from 1968 to 1982, Black and White adults had similar mortality rates. In the later period, from 1982 to 2010, Black adults had a significantly higher mortality rate than White adults. Within the last decade, the racial mortality gap has decreased for both sexes. However, after deconstructing the trends into rural and urban settings, the increasing racial mortality gap for men in rural counties, which started in the early 1980s and increased thereafter, indicates the need for additional research to understand the factors associated with these disparities. A study by Case and Deaton^1^ indicated a decreasing racial mortality gap in the younger population (age 25-54 years). Using unadjusted mortality rates, Cunningham^2^ reported a similar decrease in the racial mortality gap among the older population (age ≥65 years) (eFigure 4 in the Supplement). Our study of age-adjusted mortality, which was adjusted for county-level socioeconomic characteristics in the older population, is consistent with these findings, but only among adults in urban areas, in which we found decreasing racial disparities in mortality rates.

After adjusting for county-level socioeconomic characteristics among a subsample of the study, the mortality rate trends indicated that the racial gap remained substantial for men residing in rural areas, but the racial gap decreased for women. After controlling for county-level income and unemployment rates, the rural vs urban disparities in mortality decreased among White men and among women of both races but remained substantial among Black men. This finding suggests that the socioeconomic differences between rural and urban areas are associated with the disparities in mortality rates among women of both races but not among Black men.

The rural and urban trends in the age-adjusted mortality rates of older adults in the US were consistent with the trends of mortality in the general US population.^2,3^ The rural-urban mortality gap in the general population has been associated with unintentional injuries, cardiovascular disease, chronic obstructive pulmonary disease, and lung cancer, which have been reported to account for 70% of the overall rural-urban gap in mortality.^3^ In terms of racial disparities, the data suggest that older Black adults living in low-income neighborhoods have higher rates of cardiovascular-associated mortality compared with older White adults living in similar conditions.^10^ In addition, Black adults have higher rates of cardiovascular mortality^11^ and die of cancer, kidney disease, stroke, and cardiovascular disease at higher rates^12^ than White adults. Cardiovascular risk factors may also be associated with higher mortality among individuals living in rural areas owing to lower access to health care, specifically to specialty care(such as cardiology, endocrinology, neurology, and inpatient rehabilitation), and the closure of hospitals in rural areas.^13,14^

Despite long-term reductions in US stroke mortality rates, decreases have slowed in the past decade, as they had in the period from 1970 to 1980. During our study period, stroke was among the 4 leading causes of death in the general US population.^15^ However, the stroke-associated mortality rate among Black adults was twice that of White adults; in contrast, the age-adjusted mortality rate for stroke was higher in Black men compared with Black women.^16,17^ Thus, the increase in mortality among Black adults in the 1980s and 1990s could be associated with the increase in stroke incidence^16,18^ and heart disease–associated deaths during this period.^19^ Furthermore, a higher incidence of disabling chronic conditions was observed among Black adults compared with White adults,^20,21^ which likely required increases in health care services; however, rural residents have limited access to emergency services,^22^ health care, health insurance, and follow-up care after hospital discharge and are more likely to have a lower socioeconomic status compared with urban residents.^23^ Thus, limited access to health care services in rural areas may be associated with increases in racial disparities in health outcomes and mortality in these communities. These increases in racial disparities may also be associated with an aging rural population^24^ with a higher prevalence of chronic disease,^4^ a shortage of primary care practitioners,^25^ and the closure of a large number of rural hospitals within the last 2 decades. More than 200 rural hospitals closed between 1990 and 2000,^13^ and an additional 160 rural hospitals closed from 2005 to 2019.^14^ Additional associations may include the risk of encountering health care stereotypes and racial and age discrimination, which may be more pronounced in rural health care settings.^26,27^

Understanding the intersectional factors of health disparities can help to inform public health, health care, and clinical interventions. For example, understanding the age, sex, racial, and geographic factors associated with better health outcomes can provide the information needed to tailor strategies at the policy, organization, practitioner, and individual levels in the service of improving health equity.^28,29,30^

### Strengths and Limitations

This study has several strengths. To our knowledge, the study, which spanned 49 years, is the longest study of all-cause age-adjusted mortality among an older US population. In addition to the long-term trend analyses of all-cause age-adjusted mortality, the decomposition of race and sex differences in rural vs urban mortality rate disparities was an important feature of this study. In comparison with previous studies that used unadjusted rates to examine racial or rural vs urban disparities in mortality rates, we used age-adjusted mortality rates to study intersectional factors in disparities, including geographic location (rural vs urban residence), race (Black vs White ancestry), and sex. Examination of national mortality data over longer periods yields greater statistical power to assess long-term trends in mortality rates.

Another important feature of this study is its examination of trends in mortality rates after adjusting for county-level socioeconomic characteristics in a subsample of the study. After controlling for socioeconomic characteristics at the county level, the adjusted trends indicated that the racial gap remains substantial for men residing in rural areas but is decreasing for women in rural areas. Furthermore, the rural vs urban disparities in mortality remain substantial for Black men, are decreasing for White men, and are almost nonexistent for women of both races after controlling for county-level socioeconomic characteristics.

The study also has several limitations. First, this study type cannot establish a causal relationship between mortality rate, rurality, and race and can only inform a statistical association. However, the study used a national county-level longitudinal design, which generated findings that allowed us to infer associations more accurately than would a cross-sectional design. Second, this study compared mortality rates between Black and White adults only. Trends for other racial populations were not included because other racial and ethnic categories were not consistently reported for the entire study period. Because we used national data, racial misclassification could have been possible, although the coding of race in the CDC WONDER Compressed Mortality Files has been found to be reliable for Black and White individuals.^2^

Third, our trend analysis is based on urban, rural-adjacent, and rural-nonadjacent county differences. An analysis using the 9 more detailed categories of the Rural-Urban Continuum Codes might indicate a different trend or greater disparities in mortality according to urbanization level. Fourth, our mortality and socioeconomic measures comprised county-level mortality, per capita income, and unemployment rates, which could vary greatly across census tracts within a given county. Because of the lack of census-tract geocodes, we were not able to analyze national mortality data at a geographic level smaller than the county.^5,31,32^ Given the compositional heterogeneity of counties, the association of poverty level with mortality reported in this article is likely to be underestimated for both urban and rural areas.^3^

## Conclusions

Overall, racial disparities in mortality rates have decreased in the last 49 years among both sexes. However, racial disparities in mortality rates increased among individuals living in rural areas after 1980 and have continued to increase, with a considerable increase in disparities observed between White and Black men living in rural-nonadjacent counties. Adjusting for county-level income and unemployment rates indicates that socioeconomic differences between rural and urban areas are associated with disparities in mortality rates among women of both races. However, among men, rural vs urban disparities remain significant, especially among Black men. Notably, the current mortality rate of Black men living in rural areas is similar to that of White men living in urban and rural areas in the mid-1980s. Controlling for income and unemployment rate, the current adjusted mortality rate of Black men in rural areas is higher than that of White men more than 2 decades ago.

Our results highlight the importance of understanding the trends in mortality rates by race, sex, and geographic area. These data can be used by policy makers to understand intersectional factors in mortality disparities as a starting point for public health and clinical interventions aimed at reducing disparities in rural regions, especially among older Black adults.

## Notes

Supplement. **eTable.** County-Level Population and Socioeconomic Characteristics, 1992 and 2014 **eFigure 1.** US County-Level Population Characteristics Over Time **eFigure 2.** Changes in US County-Level Socioeconomic Characteristics Over Time **eFigure 3.** Gap Between Rural and Urban Mortality Rates **eFigure 4.** All-Cause Unadjusted Mortality Rates by Race for Adults 65 Years and Older, 1968-2016
