# Supplementary material for: Assessment of Racial Disparities in Mortality Rates Among Older Adults Living in US Rural vs Urban Counties From 1968 to 2016
Source: JAMA Netw Open. 2020 Aug 3;3(8):e2012241. doi: 10.1001/jamanetworkopen.2020.12241 (PMC7399752; doi:10.1001/jamanetworkopen.2020.12241)
Supplement: Supplement. — eTable. County-Level Population and Socioeconomic Characteristics, 1992 and 2014 eFigure 1. US County-Level Population Characteristics Over Time eFigure 2. Changes in US County-Level Socioeconomic Characteristics Over Time eFigure 3. Gap Between Rural and Urban Mortality Rates eFigure 4. All-Cause Unadjusted Mortality Rates by Race for Adults 65 Years and Older, 1968-2016 [file jamanetwopen-3-e2012241-s001.pdf]

Supplementary Online Content

Ferdows NB, Aranda MP, Baldwin JA, Baghban Ferdows S, Ahluwalia JS, Kumar A. Assessment of racial disparities in mortality rates among older adults living in US rural vs urban counties from 1968 to 2016. *JAMA Netw Open*. 2020;3(8):e2012241. doi:10.1001/jamanetworkopen.2020.12241

eTable. County-Level Population and Socioeconomic Characteristics, 1992 and 2014

eFigure 1. US County-Level Population Characteristics Over Time

eFigure 2. Changes in US County-Level Socioeconomic Characteristics Over Time

eFigure 3. Gap Between Rural and Urban Mortality Rates

eFigure 4. All-Cause Unadjusted Mortality Rates by Race for Adults 65 Years and Older, 1968-2016

This supplementary material has been provided by the authors to give readers additional information about their work.

© 2020 Ferdows NB et al. *JAMA Network Open*.

eTable. County-Level Population and Socioeconomic Characteristics, 1992 and 2014

	1992			2014		
	**Urban**	**Rural adjacent**	**Rural nonadjacent**	**Urban**	**Rural adjacent**	**Rural nonadjacent**
Number of counties	1,158	1,021	901	1,163	1,019	910
Population	1,042,395	42,843	28,558	1,156,197	47,778	33,176
Per-capita income	20,981	15,545	15,604	47,164	36,234	38,233
Unemployment rate, %	7.49	8.33	7.76	6.23	6.71	6.57
Population ≥ 65 years, %	12.18%	15.07%	15.30%	13.96%	17.80%	17.66%
Population female, %	59.91%	58.99%	58.45%	56.43%	54.84%	54.72%
Population black, %	8.59%	6.06%	4.35%	10.47%	5.42%	3.71%
Population other race, %	2.12%	0.57%	1.43%	5.66%	0.75%	1.83%

Note: The data is derived from the Area Health Resources Files (AHRF).

© 2020 Ferdows NB et al. *JAMA Network Open*.

eFigure 1. US County-Level Population Characteristics Over Time

A. Trends in county-level ratio of older adults

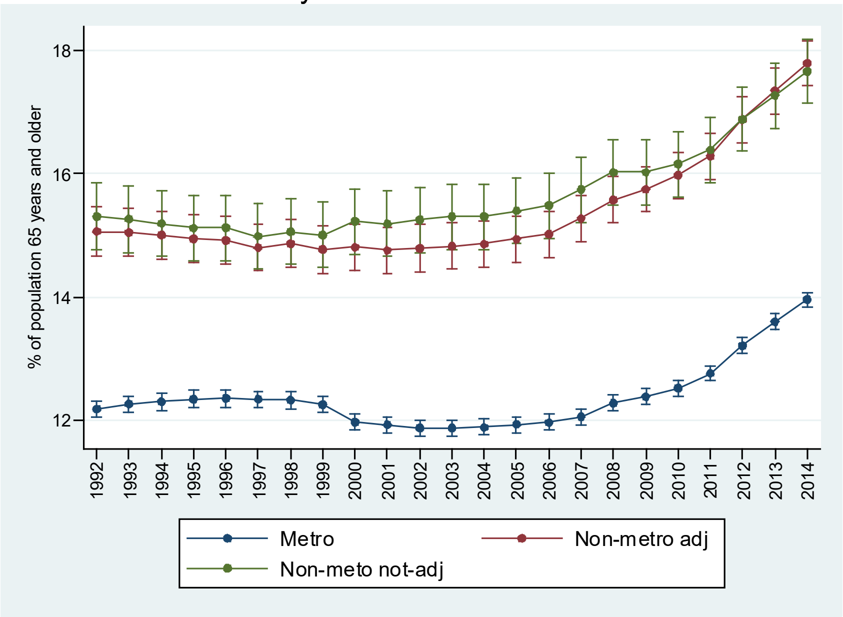

B. Trend in county-level ratio of black population

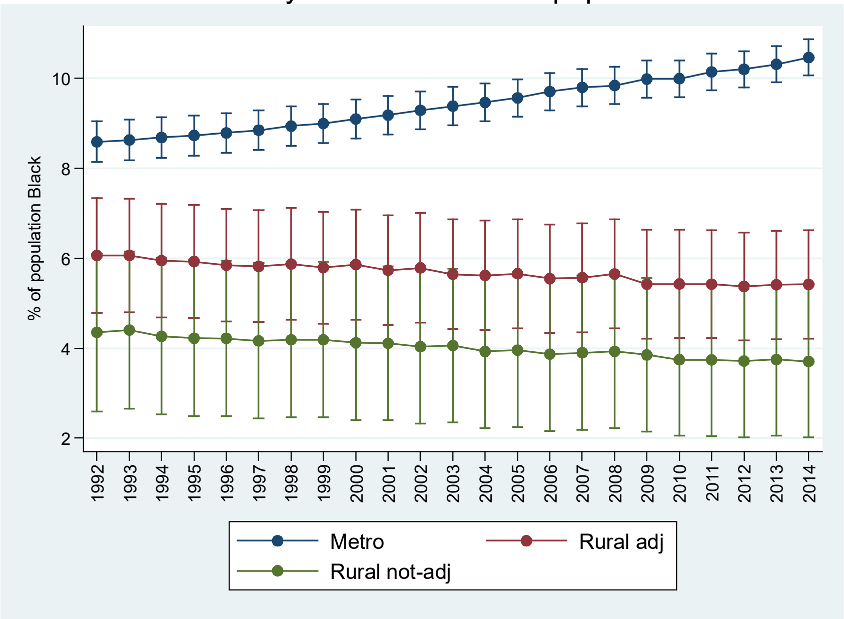

Note: Authors' calculation using data from the Area Health Resources Files (AHRF) from 1992 to 2014. All graphs are weighted by population size. The error bars represent 95% CIs.

© 2020 Ferdows NB et al. *JAMA Network Open*.

eFigure 2. Changes in US County-Level Socioeconomic Characteristics Over Time

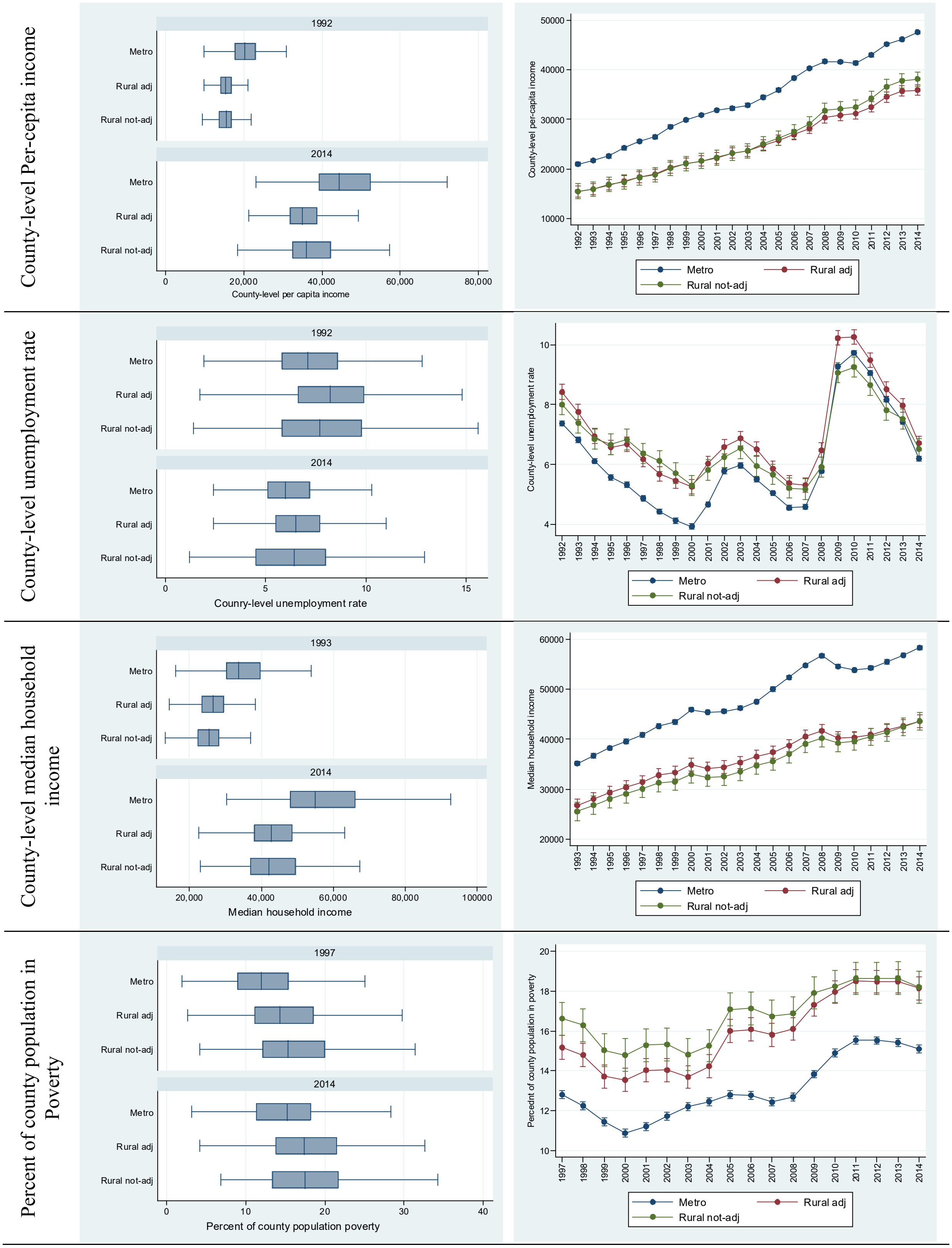

© 2020 Ferdows NB et al. *JAMA Network Open*.

Note: Authors' calculation using data from the Area Health Resources Files (AHRF) from 1992 to 2014. All graphs are weighted by population size.

© 2020 Ferdows NB et al. *JAMA Network Open*.

eFigure 3. Gap Between Rural and Urban Mortality Rates

A. Unadjusted Mortality, 1968-2016

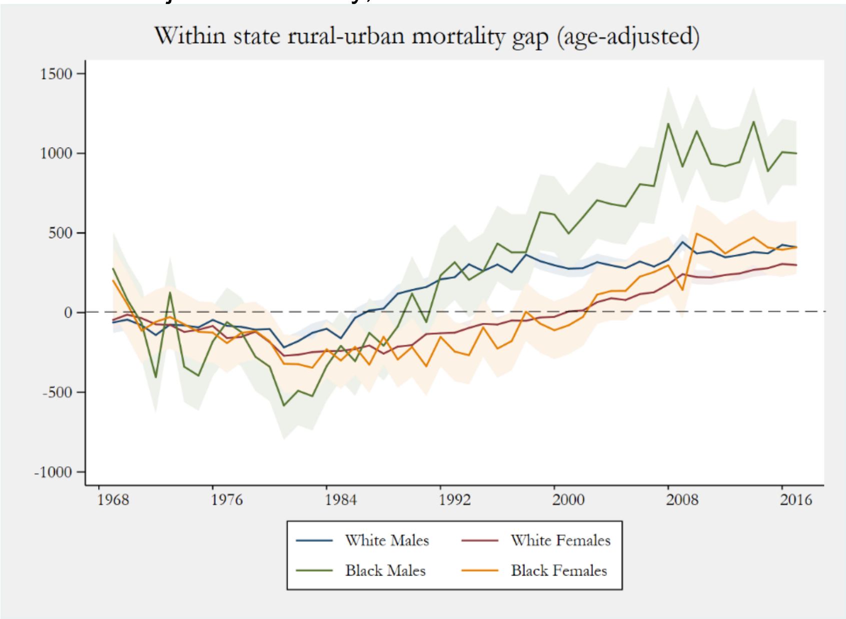

B. Adjusted Mortality, 1992-2014

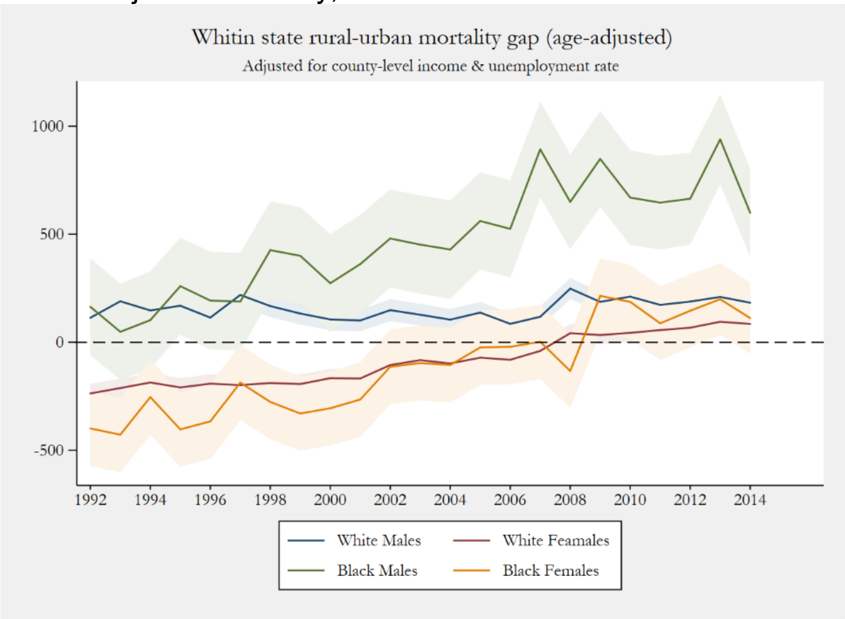

Note: All-cause age-adjusted mortality rate per 100k population 65 years and older. The data for mortality rates and county-level population characteristics was derived from CDC-Wonder compressed mortality files. The data on county-level per-capita income and unemployment rate is derived from the Area Health Resources Files (AHRF).
The plots were weighted by county population. The error bars represent 95% CIs.

© 2020 Ferdows NB et al. *JAMA Network Open*.

eFigure 4. All-Cause Unadjusted Mortality Rates by Race for Adults 65 Years and Older, 1968-2016

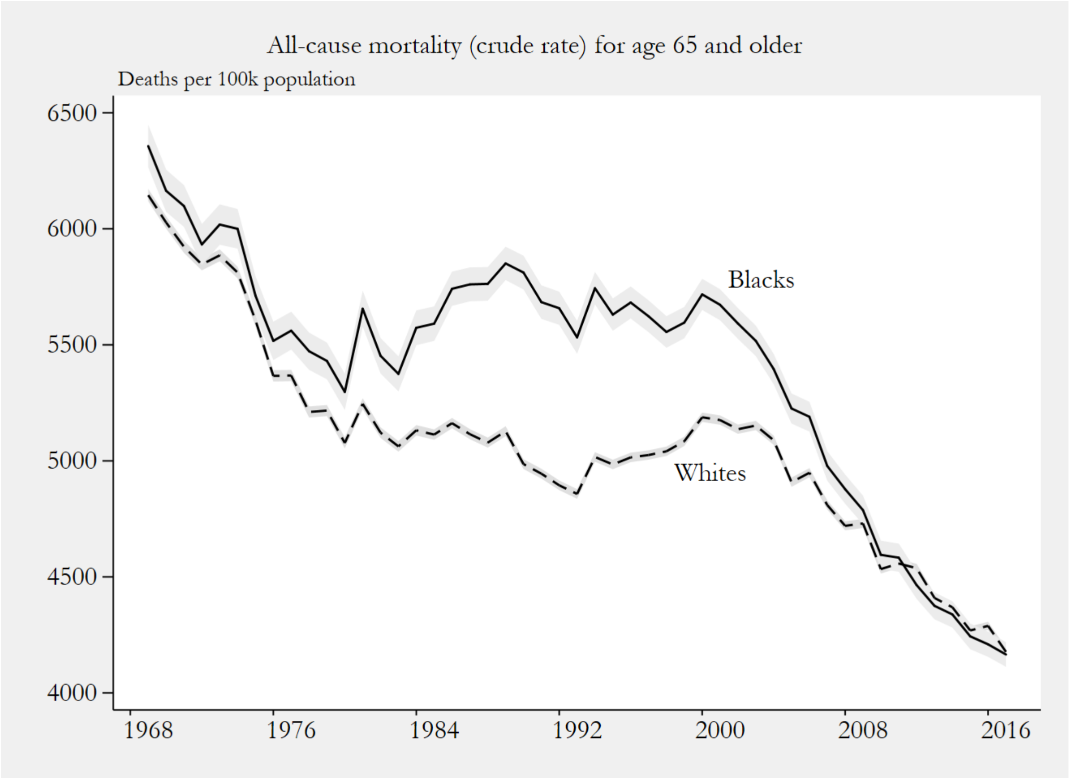

Note: All-cause mortality (crude rates) per 100k population 65 years and older. The plots were weighted by county population.

© 2020 Ferdows NB et al. *JAMA Network Open*.
